# Verbal and Cross-Modal Ratings of Music: Validation and Application of an Icon-Based Rating Scale

**DOI:** 10.1177/2041669519852643

**Published:** 2019-06-11

**Authors:** E. Schubert, M. Murari, A. Rodà, S. Canazza, O. Da Pos, G. De Poli

**Affiliations:** Empirical Musicology Laboratory, School of the Arts and Media, UNSW Sydney, Australia; Department of Information Engineering, University of Padova, Italy; Department of General Psychology, University of Padova, Italy; Department of Information Engineering, University of Padova, Italy

**Keywords:** cross-modal association, multisensory response, colour, temperature, emotion, music, maluma/takete, self-selected stimulus

## Abstract

Can music be rated consistently using nonverbal descriptors such as colours and temperatures? 144 participants rated 6 experimenter-selected and 2 self-selected pieces of music along 15 bipolar icon (graphic) scales intended to portray emotions, and sensory experiences consisting of colour, temperature, shape, speed, texture, and weight. Participants also rated the same pieces using bipolar verbal scales which aimed to encompass the concepts represented by the icons (e.g., the word “red” for the colour red). Furthermore, the icons themselves were subjected to open-ended verbal labelling to validate the icon scale. Colour icons spontaneously evoked a cross-modal association on 67% of occasions: blue being cool, and red/orange being warm or hot, and the icon scale had overall good face validity. Music regularly and consistently evoked multisensory associations (using the icon scale) including shapes, colours, weight, and temperatures, in addition to emotions. Cross-modal perception is indicative of music’s character rather than the enjoyment of the music. The icon scale provides new insights into music perception and for applications where language skill may limit participant expression.

## Introduction

Our aesthetic responses to objects are often couched in some form of verbal reports. One can describe an object, explain how it makes them feel, say what it reminds them of, and so on. But can these kinds of word-based descriptions exhaust our understanding of the aesthetic experience? A piece of music might be described consistently as expressing sadness and beauty. But might it not also be blue in colour, or heavy in weight, or feel warm in temperature? Might a stimulus such as a piece of music evoke sensations in various modalities or senses or might those modalities and senses be able to reliably describe the music? In recent years, the examination of cross-modal association has started to draw increasing interest from researchers studying responses to the arts and is promising new, exciting insights for further understanding how we process stimuli (De Poli, Murari, Canazza, Rodà, & Schubert, 2017). This study aims to extend this work by applying a recently developed icon scale to the rating of music excerpts.

Cross-modal association is interesting both for its own sake and because of the understanding it brings to behaviour, cognition, and neuroscience (Spence, 2011). Some researchers seek to identify the mechanisms and pathways responsible for cross-modal associations as well as the influence of individual differences upon them. There is limited consensus on the definition of cross-modal associations, but in the current research, it refers to a general kind of cross-modal connection that individual (with or without synaesthesia) can experience (Martino & Marks, 2001). The cross-modal connections that are particularly interesting are those that are made regularly and consistently.

Robust findings for cross-modal associations have been reported for musical features such as musical pitch with physical size (Bien, ten Oever, Goebel, & Sack, 2012), height (Cabrera & Morimoto, 2007; Küssner, Tidhar, Prior, & Leech-Wilkinson, 2014; Pratt, 1930), and thickness (Dolscheid, Shayan, Majid, & Casasanto, 2013). These connections can be explained by structural correspondences because of similarities of neural coding across modalities (for a review, see Parise & Spence, 2013). However, the more complex and rich associations that sections and whole pieces of music form are less well understood.

One study investigating cross-modal associations with music was conducted by Palmer, Schloss, Xu, and Prado-León (2013). They asked participants to select colours from a palette of 37 that go with each of 18 classical music excerpts. In a second experiment, the same task was completed for 14 facial expressions (instead of the music). In a third experiment, the task was to match each music example with faces from a set of 13 emotion faces expressing happiness, sadness, and anger. The consistency of matching across the three sets of pairing of the three domains—music, colours, and emotion faces—was taken as evidence that cross-modal matching of colour with music was mediated by emotion. For example, fast music in the major mode was rated as more yellow and happier than music that was slow and in the minor mode, which was relatively bluer and sadder. The findings of the Palmer et al. study linking music to colours and emotions have been extended to a variety of music styles and moods (Whiteford, Schloss, Helwig, & Palmer, 2018).

Studies like Palmer et al. (2013) are beginning to provide insights into the nature and substance of cross-modal associations for “real” pieces of music. But more research is needed. While testing for associations between one mode and another (such as music and colour) allows us to hone in on the specifics of colour associations with music, and if there exist any regularities, such work can only indirectly (as does the study by Palmer et al.) tell us which modes are preferentially ignited, if at all. A simple, though methodologically laborious, approach was conducted through a series of studies by De Poli et al. (2017). They found various regular relationships between music and a small selection of representative samples in different modalities. Their method required listeners to respond to music by using a variety of multimodal response options, including immersing one’s hand in cold water then warm water, and asking which sensation better described the music, then repeating the process but this time holding a soft object versus a hard object. Other intramodal based pairs used for response to the music were colours (blue–orange), visual shapes (smooth–jagged), tactile (smooth–rough), taste (bitter–sweet), and musculoskeletal/proprioceptive (heavy–light). The method produced reliable results, with Cronbach’s α (an index of the reliability of the rating scale) for seven nonverbal scales reported at .9 (Murari et al., 2014). They used a range of structurally distinct classical music excerpts which were selected because of their *a priori* expression of a range of emotions.

Several consistent nonverbal musical associations were reported. For example, in one experiment, Murari et al. (2014) found that six excerpts of music could be reliably connected to particular shapes (smooth or jagged), and colour associations were also fairly consistent, with an excerpt from the Adagio from Mozart’s piano concerto K 488 regularly reported as the colour blue, and less regularly reported using the word “blue.” These findings have been replicated (Murari, Rodà, Da Pos, et al., 2015) and have been found to produce similar results when the different modalities are represented by purely visual analogues in the form of icon pairs (Murari, Schubert, Rodà, Da Pos, & De Poli, 2017), for example, images of hard objects formed into one icon and images of soft objects formed into another icon for the “hard–soft” icon scale. The icon based nonverbal scale had the advantage of allowing a wider range and larger number of modes to be represented (e.g., instead of just warm and cold, an icon could represent heat with a fire icon and cold with an image of a blizzard, things that are difficult to realise safely and inexpensively in a laboratory). However, one possible limitation of the icon-only scale is that we cannot be sure how closely each evokes the intended modality or indeed whether they themselves evoke different modalities or emotions (e.g., heat-evoking comfort or blue-evoking coldness). Murari et al.’s (2017) study applied the icon scale to investigate responses to pieces of music, but no investigation of the validity of the scale was reported. We decided to expand that study accordingly and to further investigate the nature and regularity of cross-modal associations with music.

The overall aim of the study was to see whether music consistently evoked particular cross-modal associations using icons and words to represent the different modalities (Aim 1). We also wanted to investigate whether excerpts exhibiting a range of musical characteristics and expressing a range of emotions could be differentiated using icons representing different sensory modalities (Aim 2), and to see whether preferred music (music with a positive “hedonic tone”) ignites cross-modal associations for the listener in a systematic manner (Aim 3).

To start to address these matters, we developed a novel, two-stage experimental paradigm. In the first stage, responses made using the icon scale were applied to extracts of music. Then, in the second stage, the response item icons themselves were rated using open-ended, free response, with the aim of validating the icon scale (by examining whether expected descriptions of icons are commensurate with the intended meaning of the icon).

## Method

### Participants

One hundred forty-four participants completed the study in line with university ethics procedures: 57 were males, 85 females, and 2 did not disclose their sex. Average age was 21.45 years (standard deviation = 4.73, range 17–59). The average years of musical instrument lessons reported was 4.93 years (standard deviation = 4.77, range 0–16). Participants did not report any uncorrected problems with vision or hearing. Data for three participants were excluded because they failed a “concentration check” question (see Note d of Table 1).

**Table 1.** Affect based Verbal Items Used in Rating Scale.

	Wording of poles^a^	Abbreviated^b^
1^c^	The music is sweet—The music is bitter	sweet–bitter
2^c^	The music is relaxed—The music is tense	relaxed–tense
3	The music is very familiar—The music is very unfamiliar	unfamiliar–familiar
4	I LIKE this piece—I DON’T LIKE this piece	like–don’t like
5	The music makes me feel active—The music makes me feel passive	feel passive–active
6	The music makes me feel strong—The music makes me feel weak	feel weak–strong
7	The music makes me feel pleasant—The music makes me feel unpleasant	feel unpleasant–unpleasant
8	The music makes me feel happy—The music makes me feel sad	feel sad–happy
9	The music makes me feel scared—The music makes me feel angry	feel angry–scared
10	The music makes me feel calm—The music makes me feel excited	feel excited–calm
11^d^	The left-most, nearest dot is the one to click—This row is a concentration check	

*Note.*

a Wording of poles as presented to the participant. Participants were given the following instruction for the verbal scale (including verbal items listed in Table 3): “For each pair of statements, select the one that is best. Do this by clicking on the dot in the row of seven dots that is closest to that statement. You may click on any of the other dots in the row if you think it provides a better answer. Please give your initial impression.” with a 7-point scale separating the two poles (separated by a dash in the table).

b Abbreviated labels for verbal scales used by researchers (not seen by participants).

c Rows 1 and 2 were items intended for icon equivalents given their significance in music research (Burzynska, 2018; Knoeferle, Woods, Käppler, & Spence, 2015; Spence & Wang, 2015), but the researchers did not find sufficiently satisfactory icon combinations during piloting. We decided to retain the verbal rating items for consistency with previous studies using sensory scales.

d Row 11 was inserted at random among the verbal items for any one of the music excerpts (also selected at random). Incorrect response to this item led to deletion of responses by the participant from the data set. This deletion was performed prior to the count of participants reported. There were three such cases.

### Stimuli

The stimuli consisted of two participant- (self) selected pieces and six experimenter-selected excerpts. The instructions the participants received for the self-selected pieces were that one should be a piece that is liked and the other a piece that is disliked. They were instructed that both pieces should be available on a media streaming website, such as YouTube. The experimenter-selected pieces were taken from previous research (Bigand, Vieillard, Madurell, Marozeau, & Dacquet, 2005; Rodà, Canazza, & De Poli, 2014, also used in the Murari et al. studies reviewed above) used to evoke a wide range of emotions (for a description, please see appendices in Bigand et al., 2005) and consisted of a variety of combinations of mode (major or minor) and tempo (fast or slow). The details of the experimenter-selected excerpts are shown in Table 2. Those excerpts are referred to by the name of the composer in this article. The icon columns in the table refer to the results and are discussed in Analysis 3.

**Table 2.** Experimenter Selected Stimuli.

Composer and reference	Piece^a^	Mode and tempo characteristics	Duration of excerpt (in seconds)	Indicative icons^b^	Icon labels^b^
Bach	Badinerie from Orchestral Suite n. 2, BWV 1067	Minor, fast	8	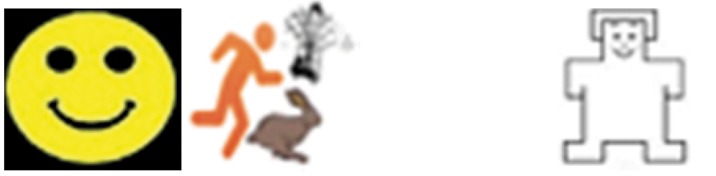	Happy face/Positive SAM, Fast
Bizet	Symphony No. 1 in C major, Allegro vivo	Major, fast	6	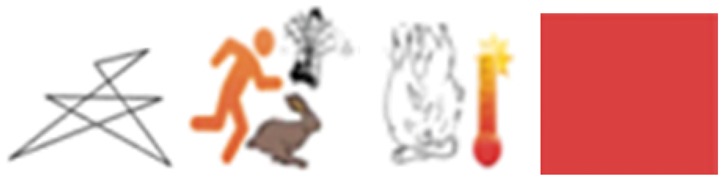	Takete/Kiki, Fast, High Arousal SAM, Hot, Red/Orange
Brahms	Violin Concerto in D major, op. 77, Adagio	Major, slow	10	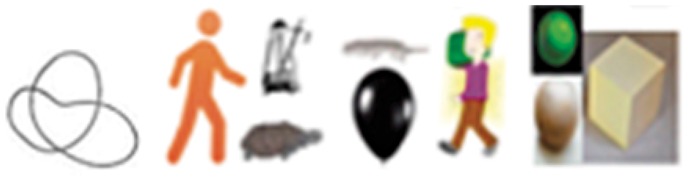	Bouba/Maluma, Slow, Sleepy SAM, Soft, Light, Smooth
Chopin	Prelude in G Minor, Op. 28, No. 22	Minor, fast	8	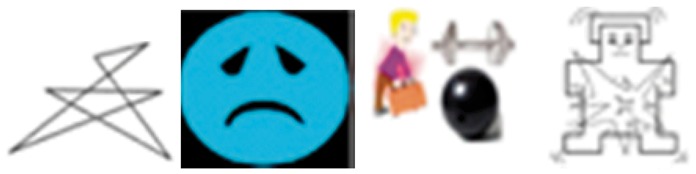	Heavy, High Arousal SAM, Kiki/Takete, Sad face
Mozart	Adagio, Piano Concerto No. 23 in A Major, K 488	Minor, slow	19	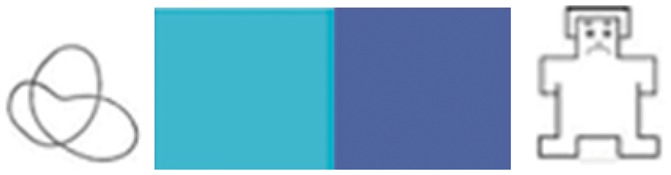	Slow, Maluma/Bouba, blue/cyan, scared, calm, sad faces, Negative SAM
Vivaldi	Allegro, Trio Sonata in C major RV82, Allegro	Major, fast	8	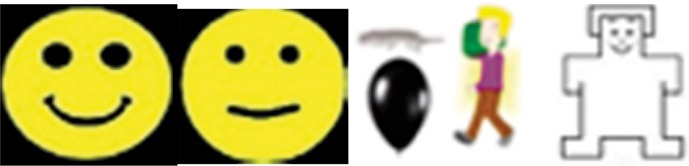	Happy/calm face, Positive SAM, Light

*Note.* SAM = Self-Assessment Manikin; BWV = Bach Werke Verzeichnis.

a All excerpts commence at the opening of the movement indicated, with the exception of the Brahms which commences at the opening of the oboe solo.

b Up to the four most representative (indicative) icons from bipolar icon rating items and with Cohen’s *d* > 0.8 are shown. The indicative icons column shows the icons taken from the representative pole; the icon label column is the verbal reference to the icons used by the researchers, listed roughly in descending order of effect size for each stimulus. For further details, see Analysis 1 below, and Supplemental Material Table 2. Icons that express similar meaning—such as some of the emotion related icons, some of the colour icons, and some of the shape icons—are grouped together to conserve space. Note that the Maluma/Bouba icons were always rated similarly, as were the Takete/Kiki icons, but only one from each pair is shown in the Indicative icons column to conserve space. Also note that Maluma and Bouba icons are labelled as such arbitrarily to differentiate the two similar looking icons, the same being the case for Takete and Kiki labelled icons. That is, there is no particular reason to call one icon Maluma and the other Bouba. See Note a of Table 4 for technical details of colours used.

### Material

The icon response scale developed by Murari et al. (2017) was used for cross-modal analogue data collection in this study (Table 3). The development of that scale was based on the graphic differential technique which has origins in work by several researchers (Espe, 1985; French, 1977; Hevner, 1935), with an important contribution by Osgood (1960) who in addition to identifying advantages in using such a scale with participants lacking the skills to read the language in which the instrument is presented, also found that the items grouped into the well-documented dimensions of meaning commonly referred to as Evaluation, Potency, and Activity (Osgood, Suci, & Tannenbaum, 1957) or valence, dominance, and arousal, respectively (Bradley & Lang, 1994). The extant graphic scales each had limitations. In the cross-cultural study developed by Espe (1985), some graphic differential scales were “problematic” when tested on a group of German students. According to Osgood (1962),

the highly generalized nature of the affective reaction system—the fact that it is independent of any particular sensory modality and yet participates with all of them—appears to be the psychological basis for the universality of three factors of Evaluation, Potency, and Activity, as well as the basis for synesthesia and metaphor. (p. 21)

**Table 3.** Comparison of Multisensory Verbal and Icon Rating Items Used in Rating Scale.

	Verbal items		Icon items		
	Wording of poles^a^	Abbreviated^b^	Icons^c^	Most frequent^d^	Labels^e^
1	The music is takete— The music is maluma	takete–maluma	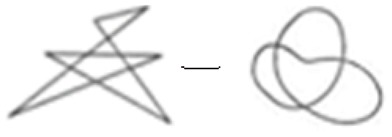	sharp–smooth	Takete–Maluma
2	The music is kiki— The music is bouba	kiki–bouba	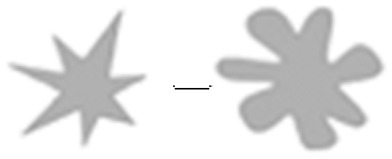	sharp–smooth	Kiki–Bouba
3	The music is orange— The music is blue	orange–blue	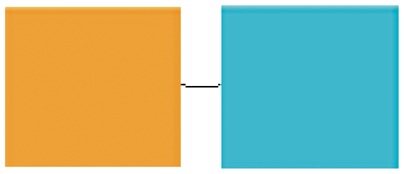	orange–**cold**/blue	Orange–Cyan
4			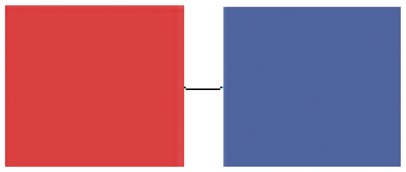	**warm**/red–blue	Red–Blue
5	The music is rough— The music is smooth	rough–smooth	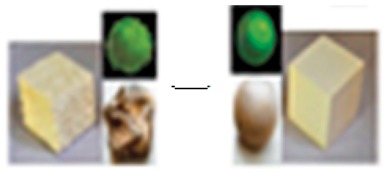	rough–smooth	Rough–Smooth
6	The music is warm— The music is cold	warm–cold	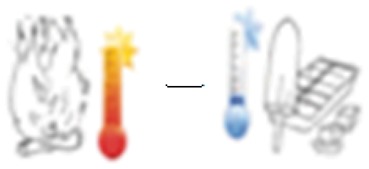	hot–cold	Hot–Cold
7	The music is hard— The music is soft	hard–soft	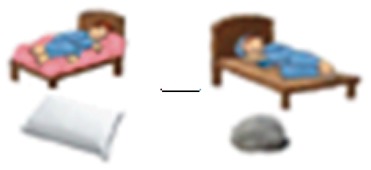	hard–**comfortable**	Hard–Soft
8	The music is heavy— The music is light	heavy–light	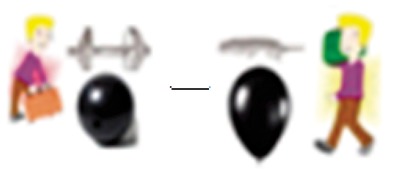	heavy–light	Heavy–Light
9			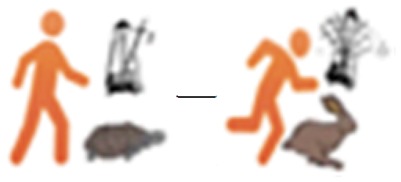	slow–fast	Slow–Fast
10	The music is active— The music is passive	active–passive	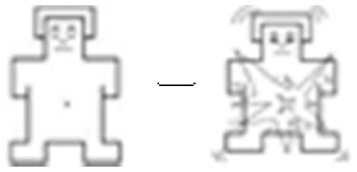	excited–calm	SAM aroused–sleepy
11	The music is pleasant— The music is unpleasant	pleasant–unpleasant	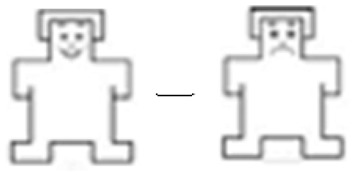	happy–sad	SAM pleasant–unpleasant
12	The music is strong— The music is weak	strong–weak	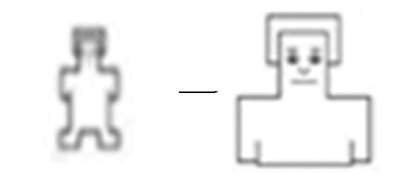	**big–small**	SAM dominant—submissive
13	The music is angry— The music is scared	angry–scared	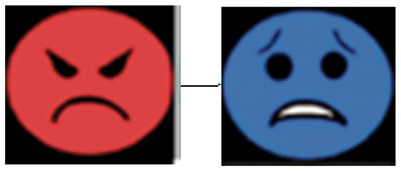	angry–sad	Face pair: angry–scared
14	The music is sad— The music is happy	sad–happy	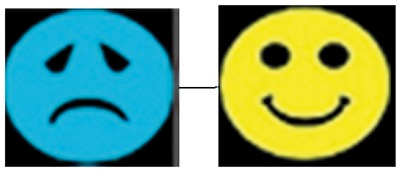	sad–happy	Face pair: sad–happy
15	The music is excited— The music is calm	excited–calm	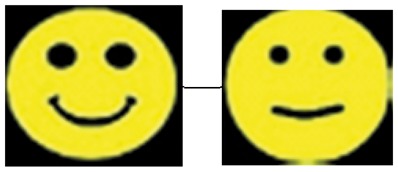	happy–neutral	Face pair: excited–calm

*Note.* SAM = Self-Assessment Manikin.

a Wording of poles read by participant. Order of presentation of pairs of poles is arbitrary in the table.

b Abbreviated labels for verbal scales as used by researchers. For verbal scale items that do not have icon versions, see Table 1.

c Icons shown at each pole. Participant read the following instructions for the icon rating items:

“ For each pair of images, select the one that best reflects the character of the music (i.e., what the music seems to be conveying or representing or expressing). Do this by clicking on the dot in the row of seven dots that is closest to that image. You may click on any of the other dots in the row if you think it provides a better answer. Please give your initial impression,” with a 7-point scale separating the two poles (shown separated by a dash in the table).

d Most frequent, spontaneously reported word for icons at each pole (separate by a dash). If a cross-modal word was most frequent, the next most frequent word is also reported, the two words separated by a slash. “Unexpected” most frequent words are shown in bold font. The words are based on the words associated with each icon, as explained in the spontaneous decoding stage of the procedure. See Analysis 1 and Supplemental Material Table 2 for further details.

e Labels for icons used by researchers.

Scales from different sensorial domains (visual, haptic, gustatory, tactile, and emotion-iconic) may provide more specific ways to investigate perceptual aspects of synaesthesia and cross-modality in a low-dimensional space. The scale constructed by Murari et al. (2017) works toward resolving of these issues.

Emotions were represented using two sources: simple emoticon-like faces as used by Ferguson, Schubert, Lee, Cabrera, and McPherson (2013) and the Self-Assessment Manikin (henceforth “SAM”; Bradley & Lang, 1994). Object shapes were selected from the well-documented multisensory experiences reported for sharp versus smooth shapes and correspondingly sharp and smooth graphemic and phonemic representations: takete or kiki for sharp and maluma or bouba for smooth (Köhler, 1929; Ramachandran & Hubbard, 2001). Other icons were constructed by Murari et al. intended to represent colours (red, blue, orange, and cyan—see Note a of Table 4 for technical colour specifications), temperature (hot and cold), speed (fast and slow), texture (rough, smooth, soft, and hard), and weight (heavy and light; see Supplemental Material Table 1 and Murari et al., 2017 for further details).

**Table 4.** Summary of Statistically Significant Cross-Modal Descriptions of Icons.

Icon^a^	Cross-modal terms^b^	Cond^c^	Statistics
Red [blue] red (14)	Warm (10), hot (9), angry (3)	v	χ^2^(1, *N* = 40) = 11.774, *p* < .001, φ = 0.543**^d^
Red [blue] red (6)	Warm (12), hot (10), angry (5)	I	χ^2^(1, *N* = 42) = 19.553, *p* < .001, φ = 0.682**^d^
Orange [cyan] orange (21)	Warm (8), hot (3)	v	χ^2^(1, *N* = 34) = 1.421, *p* = .028, φ = 0.204*
Orange [cyan] orange (2)	Warm (19), harsh (3)	i	χ^2^(1, *N* = 23) = 19.696, *p* < .001, φ = 0.925**^d^
Soft [hard] comfortable (22)	Calm (4), relaxed (4), happy (3), peaceful (3)	i	χ^2^(1, *N* = 59) = 1.163, *p* = .043, φ = 0.14*
Cyan [orange] cyan (7)	Cool/cold (9), light (4), calm (3)	v	χ^2^(1, *N* = 25) = 11.878, *p* < .001, φ = 0.689**^d^
Cyan [orange] cyan (5)	Cold/cool (20), calm (8), bright (5), light (4)	i	χ^2^(1, *N* = 41) = 3.354, *p* = .001, φ = 0.286**
Blue [red] blue (16)	Cold/cool (9), sad (3)	v	χ^2^(1, *N* = 28) = 3.103, *p* = .002, φ = 0.333*
Blue [red] blue (8)	Cold/cool (15)	i	χ^2^(1, *N* = 44) = 28.823, *p* < .001, φ = 0.809**^d^

*Note.* Only word counts greater than two are shown.

**Figure 1.**
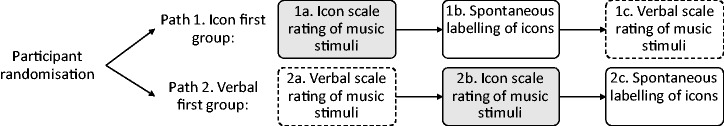
Procedural flow chart showing steps of the two-stage match and spontaneous decoding paradigm. Grey shading indicates icon condition; dashed outline indicates verbal condition.

a Icon stimulus [relative to] intramodal word (intramodal word count). For example, in row one, the colour red is presented with respect to blue, and the word (“red” in this case) is the most frequently reported intramodal word—reported 14 times. Colour icons produced significant cross-modal associations in the spontaneous labelling Blocks (1b and 2c in Figure 1) of the experiment. The hexadecimal notation RGB colour specification of the source colours used were as follows: 0000FF (blue), FF0000 (red), 00FFFF (cyan), and FFA500 (orange). Because participants completed the task on a computer of their choice, it was not practicable to calibrate display colours.

b Highest frequency cross-modal terms (count). For example, in Row 1, the colour red (when compared with blue) is described with cross-modal terms “warm” on 10 occasions, “hot” on 9 occasions, and “angry” on 3 occasions.

c Condition: v “verbal first,” i “icon first.” Refer to Figure 1, Blocks 1b and 2c.

d Effect size >0.5.

* *p* < .05. **p < .001.

In addition, a verbal rating questionnaire was constructed so as to reasonably match the meaning of the icon items. Words were also used to gather data on affective and aesthetic responses, including the rating of emotions. The latter sets of items were based on the listening appraisal sheet used by Schubert (2010). The verbal rating item pairs are tabulated in Table 3 (lower case in the table) along with the icon rating items (capitalised in the table). The use of verbal and icon rating scales would allow investigation of the validity of the icon scale. Additional verbal items, which were either alternative words for icons or without icon equivalents, were also used in the verbal scale, and these are listed in Table 1.

### Procedure

Participants read a Participant Information Sheet and signed a consent form prior to the commencement of the study. Ethics approval was provided by the host institution. Participants received course credit in return for participating. They were asked to have ready two pieces of music, one liked and one disliked, which they would use in the study when prompted. They were also asked to paste the URL (e.g., from YouTube) and provide details of the liked and disliked pieces when they reached that point of the study. They completed the study online and were requested to find a location which was quiet, where they would not be disturbed, and where they had a good audio system (speakers or headphones). The survey was designed and distributed using Keysurvey (WorldApp, http://www.keysurvey.com/). The design and procedure of the study are represented in the flow chart shown in Figure 1. Prior to experimental path selection (see Figure 1), demographic information was requested, and participants were randomly assigned to the icon first group (Path 1 in Figure 1) or the verbal first group (Path 2 in Figure 1). They completed the relevant listening tasks for each piece. The pieces (including the liked and disliked pieces) were presented in a different random order for each participant. The poles of each rating item were swapped across sessions so that an item with poles A–B (e.g., Cyan–Orange) was on some occasions presented in the order A–B (left to right) and at other times B–A (left to right, e.g., Orange–Cyan). The distribution of the differently ordered poles was random. Immediately prior to the commencement of the first task requiring listening to music, participants were asked to check that their sound systems were working, that the volume was set to a comfortable level and to put on headphones if not listening using loudspeakers.

When ready, the participant in the icon first group commenced the icon condition (Block 1a in Figure 1, see Murari et al, 2017 for analysis of data collected in this block). The participant clicked a start button for the experimenter selected pieces, and in a new window commenced playing a YouTube (or similar) link before returning to the experiment window of the browser. This triggered audio playback, and the particpant commenced listening to a piece of music, and rated an icon pair item that best matched the music for each of the 15 icon pair rating items. Instruction wording is quoted in Note c of Table 3. When participants had completed their responses to a piece of music, they could not return to check their answers and moved on to the next piece.

At the end of the icon response condition, participants were shown a list of icons, being each of the thirty icons (Table 3) used in the icon response questionnaire (Block 1b in Figure 1). This was the spontaneous decoding task described earlier. Participants were asked to describe each icon using just one or two words. To make the responses interpretable with respect to the icon rating of music task just completed, participants were asked to make the description of the icon in comparison to the icon used as its polar opposite pair. For example, when asked to provide a word or two to describe the blue square icon, the participant was told to do it relative to the red square icon (the same icon with which it was paired in the icon scale rating of music stimuli just completed). Both the target (to be labelled) and the comparison (with respect to) icons were shown.

At the end of the spontaneous decoding stage participants in the icon first condition were encouraged to rest for a couple of minutes and then continue with the next task which was the verbal response task (Block 1c in Figure 1). The participants listened to the same excerpts as in Block 1a again, in a different random order and completed the rating items for each excerpt using the verbal rating items. Instruction wording is quoted in Note a of Table 1. Poles of the verbal rating items were swapped across participants using the same scheme as described for the icon rating items. Participants assigned to verbal first group (Path 2 in Figure 1) were invited to rest at the end of that stage, before commencing with the icon condition (Block 2b in Figure 1) followed by the spontaneous icon decoding task (2c).

## Results and Discussion

### Analysis 1: Verbal Validation of Icons

The first analysis aimed to test whether icons were labelled according to the researcher-expected concept and to then see which, if any, participant reported labels used terminology that was cross-modal. Data preparation of spontaneous free report terms to describe icons was based on the procedure adopted by Augustin, Wagemans, and Carbon (2012). Spelling errors were corrected, articles for nouns and qualifiers were removed, and different spellings and same-stemmed words were pooled. The word count was conducted separately for the icon first group (using responses from Block 1b in Figure 1) and the verbal first group (using responses from Block 2c). This way any systematic influences due to context of the verbal first condition upon labelling icons could be assessed. That is, we could investigate whether performing the word rating task first primed the words used for the icon labelling differentially to the icon first condition (when icons were labelled before any lists of words were presented). Words for each icon in each condition were tallied. A list of all the icons showing most frequent words encountered for each icon by condition in descending order is shown in Supplemental Material Table 1. The first of each triad of rows shows the results for the two conditions combined, followed in the subsequent rows by the icon first and then the verbal first group responses. The table also groups the responses into emotion-related (top rows), colours (Red, Blue, Cyan and Orange), temperature, shapes, speed, texture, and then weight.

Inspection of the frequency table (Supplemental Material Table 1) demonstrates overall congruence between intended icon meaning and the most frequently reported words used to spontaneously describe the icon, as shown in Table 3, with three exceptions: the Soft icon, the SAM dominant–submissive icons, and the colour icons. The Soft icon was most frequently reported as “comfortable,” which is semantically related to “soft,” but takes the perspective of the individual in contact with the icon (a person in a bed with pillow and mattress), rather than the tactile sensation itself. The SAM dominant – submissive icons were both most frequently described in terms of their relative physical size (dominant icon described as big, and Submissive icon submissive as small), suggesting the physical appearance of the icons rather than affective portrayal. Because this result seems to be a description of physical resemblance, rather than the researcher-intended meaning, its application in emotion research and this study should be carried out with caution.

The frequently reported, unexpected descriptions of colours require special attention. Specifically, in the icon first group (where verbal priming for icon labels was not present), the Blue colour icon was reported as calm more frequently than any other word (16% of total word count). This was followed by cold (14%, also cross-modal) and then blue (12%, intramodal). This was somewhat different in the verbal first group, where the most frequently reported word for the Blue icon was intramodal (blue, 31%). This finding suggests that the participant reading the *word* “blue” in the earlier part of the study may have primed activation of the colour which was then easier to activate later in the study when the actual colour was presented, inhibiting activation of cross-modal activation of the colour (such as cold) to some extent (Collins & Loftus, 1975). Still, even then, the second most frequently nominated word *was* of a cross-modal term (cold, representing 13% of words used to describe that icon in that verbal first condition). The icon colour orange was frequently reported as warm (18% in icon first condition and 16% in verbal first condition), although orange was the most frequent verbal descriptor in the verbal first condition (40%). Cyan was reported as cold in the icon first condition (14%). According to χ^2^ tests conducted for each of the icons that consisted of frequent cross-modal descriptions (testing whether occurrence of intermodal term occurred above the proportion of intermodal items occurring across the pooled distribution of all responses: see Augustin et al., 2012, for further details about this method), all four colour icons produced a significant number of cross-modal verbal descriptions (Table 3). With all colour responses pooled, 67% of spontaneously reported words were cross-modal. This unexpected high frequency of spontaneous cross-modal description of colours reflects a somewhat extreme case of known cross-modal association between colour and temperature (Da Pos & Valenti, 2007; Osgood et al., 1957). But this is the only study to our knowledge where the colour–temperature association has been demonstrated by spontaneous, free verbal labelling. Furthermore, given that the temperature icons were not decoded as colours, strengthens the argument proposed by Lorentz, Ekstrand, Gould, and Borowsky (2016) that colour–temperature cross-modal processing direction is primarily from colour to temperature rather than temperature to colour. In the present context, this finding is even more impressive given that emotions were being judged in other parts of the experiment, and colour–emotion (Fetterman, Robinson, & Meier, 2012; Mentzel, Schücker, Hagemann, & Strauss, 2017) or temperature–emotion (Ijzerman & Semin, 2009; Wilkowski, Meier, Robinson, Carter, & Feltman, 2009) assocations may well have been activated preferentially to colour–temperature connections alone but were not. Therefore, cross-modal correlations between temperature and another modality (such as music) may be mediated by colour rather than emotion (etc.). The only other frequently mentioned cross-modal verbal description was calm (10%) in describing the Slow icon (in the verbal first condition). However, ordering of conditions does not appear to have played a major role in the words used to spontaneously decode icons, and the overall consistency of words used to describe the icons support the face validity of the icon scale, suggesting that the instrument could be used to assess music.

### Analysis 2: Factor Analysis of Icon and Verbal Ratings

To address Aim 1 (could music consistently evoke associations cross-modally), a factor analysis was conducted using the pooled data (icon-first and verbal-first, i.e., Blocks 1a, 1c, 2a and 2b in Figure 1). The analysis of the combined data revealed three interpretable factors, as shown in Table 5. The first two factors depicted the concepts that we labelled valence and arousal, each with a balance of items loading from the icon item set and the verbal item set (denoted with an equal sign after the item pole label in which there was similarity across the modalities). The frequent loading of verbal and plausibly corresponding icons for the arousal labelled dimension suggests that icons can be used instead of verbal scales for collecting responses to music (Aim 1), further supporting the finding of good agreement in the verbal labelling of icons reported in Analysis 1.

**Table 5.** Three-Factor Analysis Loading Matrices for Both Groups (Icon First and Verbal First).

	Factor loadings			Item poles	Factor label and dominating item pole for factors		
	1	2	3		Arousal	Valence	Valence (verbal)
Icon items	0.766	0.069	0.102	SAM aroused–sleepy	aroused=		
	−0.062	0.813	0.172	SAM unpleasant–pleasant		unpleasant	
	0.460	−0.106	−0.013	SAM dominant submissive	dominant=		
	0.531	−0.092	0.263	Face pair: angry–scared	angry=		
	−0.105	0.858	0.098	Face pair: sad–happy		sad=	
	0.131	−0.725	−0.151	Face pair: excited–calm		calm	
	0.784	0.145	0.087	Takete–Maluma	takete=		
	0.549	−0.071	0.173	Orange–Cyan	orange=		
	0.607	−0.116	0.210	Red–Blue	red		
	0.460	0.223	0.205	Rough–Smooth	rough=		
	0.628	−0.217	0.072	Hot–Cold	hot		
	0.455	0.542	0.152	Hard–Soft		hard	
	0.528	0.479	0.074	Heavy–Light	heavy=		
	−0.715	0.160	−0.063	Slow–Fast	fast		
	0.787	0.164	0.090	Kiki–Bouba	kiki=		
Verbal items	0.508	0.017	0.082	takete–maluma	takete=		
	0.462	−0.349	0.074	orange–cyan	orange=		
	−0.815	−0.253	−0.068	soft–hard	hard		
	0.741	0.230	0.239	rough–smooth	rough=		
	−0.382	−0.632	−0.254	sweet–bitter		bitter	
	−0.584	−0.429	−0.025	heavy–light	heavy=		
	0.002	−0.643	−0.196	warm–cold		cold	
	−0.707	−0.384	−0.046	relaxed–tense	tense=		
	−0.536	−0.022	−0.020	bouba–kiki	kiki=		
	−0.738	0.190	0.167	passive–active	active=		
	−0.654	−0.007	0.381	weak–strong	strong=		
	0.273	0.453	0.676	unpleasant–pleasant			unpleasant
	0.006	0.133	0.243	unfamiliar–familiar			
	0.182	0.265	0.760	like–don’t like			don’t like
	−0.262	0.731	0.152	sad–happy		sad=	
	0.455	0.052	0.078	angry–scared	angry=		
	−0.693	0.224	0.292	feel passive–active	feel active=		
	−0.560	0.219	0.489	feel weak–strong	feel strong=		
	0.163	0.443	0.685	feel unpleasant– unpleasant			feel unpleasant
	−0.250	0.644	0.364	feel sad–happy		feel sad=	
	0.322	0.036	0.313	feel angry–scared			
	0.788	−0.137	0.000	excited–calm	excited=		
	0.712	−0.113	−0.026	feel excited–calm	feel excited=		

*Note.* See Tables 1 and 3 for icons (capitalised) and verbal (lower case) item pole information seen by participants. Only loadings >.4 have dominating pole label shown within that factor column. Equal sign after a word in a factor indicates that it loaded in both its verbal and icon for that factor (e.g., the SAM dominant icon with the words angry/strong: the colour orange with the word orange) modalities using a loading criterion of .4. Rotation Method: Varimax with Kaiser Normalisation. Three factors extracted from four iterations. SAM = Self-Assessment Manikin.

The only notable exception for the valence factor was the Hot–Cold icon item which loaded onto arousal while the warm–cold *verbal* item did not. The verbal warm–cold item did, however, load onto the valence factor, but the icon item did not. This apparent anomaly could be reconciled by the imperfect matching of the warm and hot poles across modality. Also, the word “cold” may have a stronger “negative” valence meaning than images of cold objects. An image of a thermometer, ice cream and pieces of ice might not be viewed as negatively as the word cold when referring negatively to a “cold” person (see, e.g., Ijzerman & Semin, 2009; Walters, 2016).

The hard–soft verbal item loaded onto the valence labelled dimension, but the icon intending to depict the same dimension loaded onto the arousal labelled dimension. The results of spontaneous verbal labelling (Analysis 1) suggests that the meaning of this icon rating item was slightly different to what the researchers expected, with the “soft” intended icon being reported as “comfortable” instead of “soft,” and so the concept of comfort could be explained as (positive) valence related, rather than the low arousal association of the word “soft.”

Thus, a good general match appears between verbal and icon items sharing similar meanings, suggesting that carefully selected icon items could be used as an alternative to verbal ratings for responses to music. The third factor is interesting because only verbal items loaded onto it. Furthermore, these items were related to “affect-valence” (Schubert, North, & Hargreaves, 2016) terms concerned with liking and disliking the music and appear to have emerged as a result of the self-selected music stimuli which focus explicitly on liking/disliking as the selection criteria. The more specific relationships between icons and music are further examined in the following analysis in which each piece of music was investigated separately.

### Analysis 3: Characteristics of the Stimuli in Terms of Verbal Description and Icon Description

Evaluation of music items was conducted to address Aim 2, by analysing the mean rating for each bipolar scale (icon and verbal) and comparing the mean against the midpoint of the scale using single sample *t* tests and by calculating the Cohen’s *d* effect size. We then formed a profile of each piece by labelling it with the icon/word from the pole of each rating scale that exhibited a Cohen’s *d* of greater than .8 (large effect size—see Cohen, 1992). Detailed results are shown in Supplemental Material Table 2. Up to the four most representative icons fulfilling the Cohen’s *d* criteria are shown in Table 2 for ease of inspection and comparison. It was possible for us to inspect another publication for additional comparison. Brahms, Vivaldi, and Bizet were used in Experiment 1 and Mozart, Liszt, and Bach in Experiment 2 of Murari, Rodà, Canazza, De Poli, and Da Pos (2015). Since that study used sensory scale ratings, the results for those are reported here to allow comparison. It should be noted that the scales used in those studies were conducted in different sensory modalities, unlike the icon analogues of the nonvisual senses used in this study.

In this study, Bach is pleasant, light, happy, sweet, and active. Icons indicate that the piece is Fast, Pleasant (SAM), and Happy (face). There is a clear connection between the emotion terms across the verbal and sensory domains. In Murari, Rodà, Canazza, et al. (2015), Bach was soft, smooth, sweet, light, and warm. Sweet and light (not heavy) therefore present a consistent characterisation of the piece across modalities and across the studies.

Bizet is tense, strong, pleasant, hard, happy, excited, and active. In terms of icons, it conveys High arousal, and is Takete/Kiki, Orange/Red, and Hot. The absence of anger suggests that it is possible to perceive heat without anger. But other interpretations are possible. In Murari, Rodà, Canazza, et al. (2015), the same excerpt was reported as being takete, hard, warm, and tense, representing good agreement with the exception of colours. This could be because the previous study used only one colour scale, with no red option. However, the sensorial reporting of warmth could also explain why participants in this study chose the “hot” icon, which in this study was a depiction that could be interpreted as warm as well as hot (sketches are of a small, controlled fire emanating from a pile of wood, a thermometer showing a high level in orange/red colour, and the yellow coloured sun).

Brahms is relaxed, smooth, pleasant, light, soft, calm, sweet, and passive. In terms of icons, it is Low arousal, Slow, Maluma/bouba, Soft, Smooth, and Light (not Heavy). According to Murari, Rodà, Canazza, et al. (2015), Brahms is maluma, soft, smooth, sweet, slow, light, calm, and warm. No colour ratings were found to be significant in either study, and the amount of agreement across studies is considerable.

Mozart is smooth, soft, sad, calm, blue, and passive, and in terms of icons Slow, Scared, Maluma/Bouba, Low SAM pleasantness, Low SAM arousal, Sad face, Blue (including Cyan), Passive, Cold, and Calm face. In Murari, Rodà, Canazza, et al. (2015), this was in good agreement with this study.

Chopin is tense, strong, rough, heavy, hard, excited, bitter, and active, with icons Sad face, Takete/Kiki, High SAM arousal, and Heavy. In Murari, Rodà, Canazza, et al. (2015), the Chopin piece was found to be takete, active, fast, and tense. The strong dynamics, the use of low register notes, minor key, and fast tempo of the piece accounts for the “heavy, hard” and “tense” results across the studies.

Vivaldi is characterised as pleasant, light, happy, warm, and sweet (as opposed to bitter), while the icons that characterise it are High SAM pleasantness, Light (not Heavy), and the faces Happy and Excited. In Murari, Rodà, Canazza, et al. (2015), the same piece was evaluated as being soft, smooth, sweet, and light.

The approach taken for analysis 3 was also applied to liked and disliked music selections in order to address Aim 3. The disliked piece (used in this study only) returned a large effect size only for unpleasantness. No other ratings had a large effect size, including Negative emotion faces and SAMs (e.g., Angry face, Sad face), while the liked piece, in addition to being rated pleasant, strong, smooth, warm, and sweet, was characterised by the icons Soft and High SAM pleasantness. The absence of colour item association with either of the self-selected stimuli suggests that music–colour cross-modal associations are related to properties of the stimulus, rather than personal, affective reactions although the range of colours represented in the icon ratings was small. The absence of facial emotions and SAM item poles suggests that icons related to human musculoskeletal expression may also be more concerned with musical character rather than preference.

## Conclusion

This research was driven by a need to better understand multisensory responses to music and to better enable researchers to gather nonverbal responses to stimuli. To this end, we investigated the validity of an icon scale and looked for regularities, if any, in the way icons representing a range of modalities could represent musical assessments and experiences.

The icon scale instrument was validated by demonstrating that the word spontaneously associated with each icon was closely related to the meaning and modality intended for the icon. While the validation was overall successful, a surprising finding was that highly thermoceptive cross-modal associations were made in response to colour items Blue/Cyan and Orange/Red, but not the reverse (temperature representing icons did not evoke colour associations). This unidirectional connection suggests a statistical, conditioned response that derives from seeing a red tap (faucet) and expecting hot water to come from it and seeing a blue tap and expecting cold water to come from it. The icon scale was overall validated, but some adjustment to the Soft–Hard icon is recommended.

The first aim to see whether music consistently evoked particular cross-modal associations using icons and words produced an affirmative result. Icons could be organised into two broad factors that resembled valence and arousal dimensions. In each case, the verbal labels and corresponding, intended icons were well aligned. A third dimension also emerged, indicating a more literal kind of evaluation or affective response. The absence of a potency (or dominant) dimension could be a reflection of the limited utility of this dimension in explaining variance in response to music (Eerola & Vuoskoski, 2011; Evans & Schubert, 2008). However, it is worth keeping in mind that the numerous affect related items may have skewed the analysis towards a valence-arousal based interpretation, and future studies may be advised to separate the affective items from the more obviously sensory items.

The second aim was to see whether extracts of music expressing a wide range of emotions could be characterised by icons. Icons, including abstract shapes, were consistently linked to pieces of music, with pieces exhibiting staccato articulation (adjacent pitches temporally separated) and rapid changes in register, harmony, dynamics (Bizet and Chopin in this study) being associated with sharp, jagged objects (and frequently with the corresponding verbal labels that look and sound jagged—kiki and takete), while slower, legato music (Brahms and Mozart) is characterised by the smooth, curvaceous shapes. Music examples were also described consistently according to icons representing weight (Brahms and Vivaldi were Light, Chopin Heavy), material texture (Brahms was Smooth), and colours were also used consistently, with Mozart being Blue (and Cyan), and Bizet Hot. Speed and emotion related icons were selected in a manner consistent with verbal descriptions and appropriate to the musical character (e.g., fast icon for fast tempo). The icon scale and icons in general therefore present new opportunities for assessing music in a manner resilient to the foibles of verbal scales (such as the impact on people without sufficient language skills) and may provide a new and convenient research tool for investigating cross-modal associations that otherwise require considerably more resources for testing (De Poli et al., 2017; Murari, Rodà, Canazza, et al., 2015; Murari, Rodà, Da Pos, et al., 2015).

The final aim of the study was to investigate how liked and disliked music in general was rated in terms of icons. None of the icons were rated as indicative of disliked music, and for liked music only, an emotion-based icon (Positive valence SAM) and the Soft icon (interpreted as comfort) were represented. This finding provides a clear indication that the icons used in the scale are suitable for identification of musical character but not the affect (in terms of hedonic outcome) the music imparts on the listener.

The study is limited in several respects. One of the limitations is that only classical music excerpts were selected. This was considered necessary because we wanted to use music where the emotions expressed were varied and consistent—the pieces had all been used in previous research to express a range of emotions consistently (Bigand et al., 2005). However, future research is needed to see if the findings can be generalised to other styles of music, for example, bebop, free jazz, or Stravinsky’s modernist style with their angularity in harmonic and rhythmic structures which may be more akin to kiki, takete, and angular shaped objects than is the case in the kind of music investigated here (see, e.g., Rodá, da Lio, Murari, & Canazza, 2018). Another limitation is that the participants were sampled from a single geographic area. For an icon scale to demonstrate robustness across linguistic boundaries, the study needs to be replicated in other (particularly non-English speaking) countries. A final limitation to be considered is the assumption that icon scales must have a verbal equivalent. It is worth noting that the verbal equivalents were used for the purpose of validating the icon scale. But future research may modify the icon scale to include representations in different modalities that do not necessarily have semantic verbal equivalents but still produce consistent, meaningful results (for futher discussion, see Da Pos & Albertazzi, 2010; Da Pos & Valenti, 2007).

Given the repeatable and independent contribution of different senses and modalities to music experience, it seems surprising that research on this aspect of musical and aesthetic experience has not received more attention. This study presents some important possibilities with regard to the regular prevalence of cross-modal activation. Above all, the study supports the possibility of using icon-based multisensory analogues as a reliable tool for understanding nonverbal aesthetic responses.

## Supplemental Material

Supplemental material for Verbal and Cross-Modal Ratings of Music: Validation and Application of an Icon-Based Rating Scale Click here for additional data file. Supplemental Material for Verbal and Cross-Modal Ratings of Music: Validation and Application of an Icon-Based Rating Scale by E.Schubert Empirical Musicology Laboratory, School of the Arts and Media, UNSW Sydney, Australia M. Murari, A. RodàS.Canazza Department of Information Engineering, University of Padova, Italy O. Da Pos Department of General Psychology, University of Padova, Italy G. De Poli in i-Perception
