# Supplementary material for: Verbal and Cross-Modal Ratings of Music: Validation and Application of an Icon-Based Rating Scale
Source: Iperception. 2019 Jun 11;10(3):2041669519852643. doi: 10.1177/2041669519852643 (PMC6563411; doi:10.1177/2041669519852643)
Supplement: Supplemental material for Verbal and Cross-Modal Ratings of Music: Validation and Application of an Icon-Based Rating Scale [file Supplemental_Material.pdf]

Supplementary material for ‘Verbal and Icon Ratings of Music: Validation and Application of an Icon-Based Rating Scale’

DOI: 10.1177/2041669519852643

Schubert, E., Murari, M., Rodà, A., Canazza, S., Da Pos, O., & De Poli, G. (2019). Verbal and cross-modal ratings of music: validation and application of an icon-based rating scale. *i-Perception, 10(3),* 1–21. doi:10.1177/2041669519852643

Table 1. Spontaneous decoding word counts by icon and condition (combined, verbal first, icon first).

Table 1. Spontaneous decoding word counts by icon and condition (combined, verbal first, icon first).

Icon	Icon label used in manuscript (relative to)	Condition	Total count	Most frequent cross-modal association	Words used to describe (count; cumulative percentage) in descending order of frequency. Count is shown as the first number in the parenthesis. This is followed by percentage, which is the cumulative frequency. Only words mentioned at least 3 times are shown (Icon First N = 76 participants, Verbal First N = 78 participants).
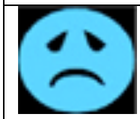	Sad (Happy)	C	124		sad (59; 48%), depressed (10; 56%), upset (9; 63%), emotional (3; 65%), unhappy (3; 68%), blue (3; 70%)
	Sad (Happy)	I	71	none	sad (27; 38%), depressed (7; 48%), upset (3; 52%)
	Sad (Happy)	V	53	none	sad (32; 60%), upset (6; 72%), depressed (3; 77%)
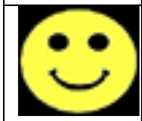	Happy (Sad)	C	106		happy (59; 56%), content (9; 64%), happier (3; 67%)
	Happy (Sad)	I	54	none	happy (29; 54%), content (3; 59%)
	Happy (Sad)	V	52	none	happy (30; 58%), content (6; 69%)
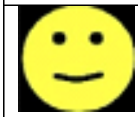	Calm (Excited)	C	128		neutral (11; 9%), content (8; 15%), sad (8; 21%), emotionless (5; 25%), calm (4; 28%), empty (4; 31%), normal (4; 34%), meh (3; 37%), mellow (3; 39%)
	Calm (Excited)	I	74	none	neutral (9; 12%), content (6; 20%), sad (4; 26%), calm (3; 30%), emotionless (3; 34%)
	Calm (Excited)	V	54	none	sad (4; 7%)
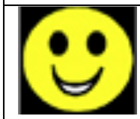	Excited (Calm)	C	103		happy (45; 44%), excited (13; 56%), super happy (3; 59%), ecstatic (3; 62%)
	Excited (Calm)	I	51	none	happy (18; 35%), excited (4; 43%)
	Excited (Calm)	V	52	none	happy (27; 52%), excited (9; 69%)
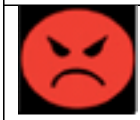	Angry (Scared)	C	98		angry (54; 55%), upset (4; 59%), mad (4; 63%), furious (3; 66%)
	Angry (Scared)	I	50	none	angry (18; 36%), mad (4; 44%)
	Angry (Scared)	V	48	none	angry (36; 75%)
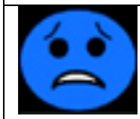	Scared (Angry)	C	139		sad (28; 20%), scared (20; 35%), worried (17; 47%), anxious (6; 51%), upset (4; 54%), fear (3; 56%), afraid (3; 58%), fearful (3; 60%)
	Scared (Angry)	I	82	none	sad (20; 24%), worried (9; 35%), scared (7; 44%), anxious (4; 49%), upset (3; 52%)
	Scared (Angry)	V	57	none	scared (13; 23%), sad (8; 37%), worried (8; 51%)
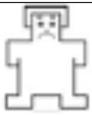	Unpleasant (Pleasant)	C	102		sad (42; 41%), unhappy (8; 49%), upset (5; 54%), disappointed (3; 57%), angry (3; 60%)
	Unpleasant (Pleasant)	I	50	none	sad (16; 32%), unhappy (4; 40%)
	Unpleasant (Pleasant)	V	52	none	sad (26; 50%), unhappy (4; 58%), upset (3; 63%)
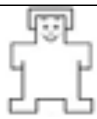	Pleasant (Unpleasant)	C	119		happy (68; 57%), excited (5; 61%), happier (5; 66%)
	Pleasant (Unpleasant)	I	68	none	happy (42; 62%), happier (4; 68%)
	Pleasant (Unpleasant)	V	51	none	happy (26; 51%), excited (3; 57%)
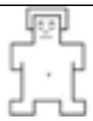	Sleepy (Aroused)	C	117		calm (33; 28%), relaxed (10; 37%), sleepy (6; 42%), peaceful (6; 47%), boring (3; 50%)
	Sleepy (Aroused)	I	61	none	calm (16; 26%), relaxed (5; 34%)
	Sleepy (Aroused)	V	56	none	calm (17; 30%), relaxed (5; 39%), peaceful (4; 46%), sleepy (4; 54%)
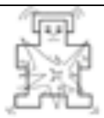	Aroused (Sleepy)	C	135		excited (11; 8%), active (5; 12%), tense (4; 15%), energetic (3; 17%), anxious (3; 19%), noisy (3; 21%), alarming (1; 87%)
	Aroused (Sleepy)	I	75	none	noisy (3; 4%)
	Aroused (Sleepy)	V	60	none	excited (9; 15%), active (3; 20%), tense (3; 25%)

Icon	Icon label used in manuscript (relative to)	Condition	Total count	Most frequent cross-modal association	Words used to describe (count; cumulative percentage) in descending order of frequency. Count is shown as the first number in the parenthesis. This is followed by percentage, which is the cumulative frequency. Only words mentioned at least 3 times are shown (Icon First N = 76 participants, Verbal First N = 78 participants).
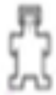	Submissive (Dominant)	C	124		small (43; 35%), insignificant (8; 41%), distant (5; 45%), tiny (4; 48%), weak (4; 52%)
	Submissive (Dominant)	I	71	Small	small (22; 31%), insignificant (5; 38%), distant (5; 45%)
	Submissive (Dominant)	V	53	Small	small (21; 40%), tiny (3; 45%), weak (3; 51%), insignificant (3; 57%)
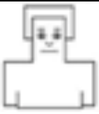	Dominant (Submissive)	C	127		big (30; 24%), large (10; 31%), close (5; 35%), bigger (5; 39%), strong (4; 43%), powerful (3; 45%), dominant (3; 47%), heavy (3; 50%)
	Dominant (Submissive)	I	68	Big	big (12; 18%), large (4; 24%), dominant (3; 28%), close (3; 32%), bigger (3; 37%)
	Dominant (Submissive)	V	59	Big/Large	big (18; 31%), large (6; 41%), strong (3; 46%)
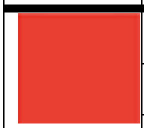	Red (Blue)	C	128		warm (22; 17%), red (20; 33%), hot (19; 48%), angry (8; 54%), intense (5; 58%), passionate (3; 60%), warmer (3; 62%), orange (3; 65%), bright (3; 67%)
	Red (Blue)	I	68	Warm/Hot	warm (12; 18%), hot (10; 32%), red (6; 41%), angry (5; 49%), intense (4; 54%), warmer (3; 59%)
	Red (Blue)	V	60	Warm/Hot	red (14; 23%), warm (10; 40%), hot (9; 55%), angry (3; 60%)
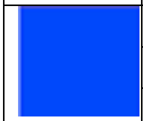	Blue (Red)	C	119		blue (24; 20%), cold (16; 34%), calm (12; 44%), cool (8; 50%), sad (5; 55%)
	Blue (Red)	I	67	Calm	calm (11; 16%), cold (9; 30%), blue (8; 42%), cool (6; 51%)
	Blue (Red)	V	52	Cold	blue (16; 31%), cold (7; 44%), sad (3; 50%)
	Cyan (Orange)	C	128		cold (15; 12%), cool (14; 23%), blue (12; 32%), calm (11; 41%), light (8; 47%), bright (6; 52%), happy (6; 56%), cooler (3; 59%)
	Cyan (Orange)	I	77	Cold	cold (11; 14%), cool (9; 26%), calm (8; 36%), blue (5; 43%), bright (5; 49%), light (4; 55%), happy (4; 60%)
	Cyan (Orange)	V	51	Cool?	blue (7; 14%), cool (5; 24%), cold (4; 31%), light (4; 39%), calm (3; 45%)
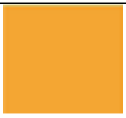	Orange (Cyan)	C	107		orange (23; 21%), warm (18; 38%), hot (5; 43%), harsh (4; 47%), angry (3; 50%), fiery (3; 52%)
	Orange (Cyan)	I	55	Warm	warm (10; 18%), harsh (3; 24%)
	Orange (Cyan)	V	52	Warm	orange (21; 40%), warm (8; 56%), hot (3; 62%)
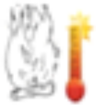	Hot (Cold)	C	127		hot (53; 42%), warm (16; 54%), angry (4; 57%), cold (3; 60%), hotter (3; 62%), passionate (3; 65%), fire (3; 67%), burning (3; 69%)
	Hot (Cold)	I	67	none	hot (25; 37%), warm (8; 49%), hotter (3; 54%)
	Hot (Cold)	V	60	Angry?	hot (28; 47%), warm (8; 60%), fire (3; 65%), angry (3; 70%)
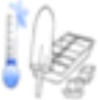	Cold (Hot)	C	109		cold (49; 45%), cool (10; 54%), colder (3; 57%)
	Cold (Hot)	I	57	none	cold (22; 39%), cool (7; 51%)
	Cold (Hot)	V	52	none	cold (27; 52%), cool (3; 58%)
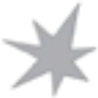	Kiki (Bouba)	C	132		sharp (25; 19%), jagged (9; 26%), spiky (5; 30%), pointy (5; 33%), rough (5; 37%), hard (4; 40%), sudden (4; 43%), fast (3; 45%), excited (3; 48%), angular (3; 50%)
	Kiki (Bouba)	I	75	Rough?	sharp (14; 19%), sudden (4; 24%), spiky (4; 29%), fast (3; 33%), rough (3; 37%)
	Kiki (Bouba)	V	57	Hard?	sharp (11; 19%), jagged (7; 32%), pointy (3; 37%), hard (3; 42%)
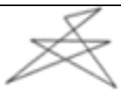	Takete (Maluma)	C	137		sharp (22; 16%), jagged (11; 24%), rough (6; 28%), complex (5; 32%), pointy (5; 36%), angular (5; 39%), spiky (3; 42%), hard (3; 44%)
	Takete (Maluma)	I	73	none	sharp (16; 22%), angular (5; 29%), rough (4; 34%), jagged (4; 40%), complex (3; 44%)
	Takete (Maluma)	V	64	none	jagged (7; 11%), sharp (6; 20%), hard (3; 25%), spiky (3; 30%), pointy (3; 34%)
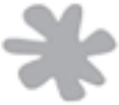	Bouba (Kiki)	C	115		smooth (22; 19%), soft (10; 28%), round (9; 36%), smoother (3; 38%), calm (3; 41%)
	Bouba (Kiki)	I	61	Soft?	smooth (13; 21%), soft (5; 30%), calm (3; 34%)
	Bouba (Kiki)	V	54	Soft?	smooth (9; 17%), round (7; 30%), soft (5; 39%)

Icon	Icon label used in manuscript (relative to)	Condition	Total count	Most frequent cross-modal association	Words used to describe (count; cumulative percentage) in descending order of frequency. Count is shown as the first number in the parenthesis. This is followed by percentage, which is the cumulative frequency. Only words mentioned at least 3 times are shown (Icon First N = 76 participants, Verbal First N = 78 participants).
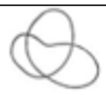	Maluma (Takete)	C	124		smooth (29; 23%), round (15; 35%), soft (5; 40%), calm (5; 44%), flowing (4; 47%), loose (4; 50%), fluid (3; 52%), rounded (3; 55%)
	Maluma (Takete)	I	65	Calm?	smooth (15; 23%), round (5; 31%), calm (4; 37%), flowing (3; 42%), loose (3; 46%)
	Maluma (Takete)	V	59	Soft?	smooth (14; 24%), round (10; 41%), soft (5; 49%)
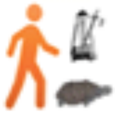	Slow (Fast)	C	105		slow (46; 44%), calm (7; 50%), fast (5; 55%), relaxed (4; 59%)
	Slow (Fast)	I	47	Relaxed?	slow (20; 43%), fast (3; 49%), relaxed (3; 55%)
	Slow (Fast)	V	58	Calm	slow (26; 45%), calm (6; 55%)
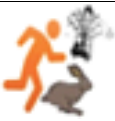	Fast (Slow)	C	139		fast (48; 35%), quick (12; 43%), faster (6; 47%), hurried (4; 50%), speedy (3; 53%), rushed (3; 55%), energetic (3; 57%), upbeat (3; 59%), frantic (3; 61%), excited (3; 63%)
	Fast (Slow)	I	80	Energetic ?	fast (27; 34%), quick (6; 41%), faster (5; 47%), hurried (4; 52%), energetic (3; 56%)
	Fast (Slow)	V	59	none	fast (21; 36%), quick (6; 46%), speedy (3; 51%)
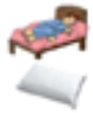	Soft (Hard)	C	148		comfortable (36; 24%), soft (22; 39%), calm (9; 45%), relaxed (9; 51%), happy (4; 54%), warm (4; 57%), peaceful (4; 59%), pleasant (4; 62%), comfy (3; 64%), comfort (3; 66%)
	Soft (Hard)	I	87	Calm?	comfortable (22; 25%), soft (11; 38%), calm (4; 43%), relaxed (4; 47%), happy (3; 51%), peaceful (3; 54%)
	Soft (Hard)	V	61	Calm?	comfortable (14; 23%), soft (11; 41%), calm (5; 49%), relaxed (5; 57%), pleasant (3; 62%)
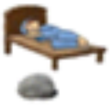	Hard (Soft)	C	109		hard (29; 27%), uncomfortable (25; 50%), cold (7; 56%), rough (5; 61%), sad (3; 63%)
	Hard (Soft)	I	54	none	hard (14; 26%), uncomfortable (13; 50%), rough (3; 56%)
	Hard (Soft)	V	55	Cold?	hard (15; 27%), uncomfortable (12; 49%), cold (5; 58%)
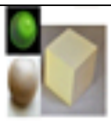	Smooth (Rough)	C	129		smooth (42; 33%), clean (7; 38%), soft (6; 43%), smoother (4; 46%), solid (3; 48%), rough (3; 50%)
	Smooth (Rough)	I	76	none	smooth (19; 25%), clean (6; 33%), soft (3; 37%), smoother (3; 41%)
	Smooth (Rough)	V	53	none	smooth (23; 43%), soft (3; 49%)
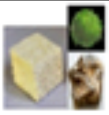	Rough (Smooth)	C	110		rough (32; 29%), sharp (5; 34%), soft (4; 37%), spiky (3; 40%), harsh (3; 43%), hard (3; 45%)
	Rough (Smooth)	I	53	none	rough (8; 15%)
	Rough (Smooth)	V	57	none	rough (24; 42%), sharp (4; 49%)
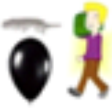	Light (Heavy)	C	121		light (43; 36%), easy (7; 41%), happy (5; 45%), calm (4; 49%), relaxed (4; 52%), carefree (4; 55%)
	Light (Heavy)	I	64	Happy?	light (23; 36%), happy (4; 42%), easy (3; 47%)
	Light (Heavy)	V	57	Relaxed/ Calm	light (20; 35%), easy (4; 42%), carefree (3; 47%), relaxed (3; 53%), calm (3; 58%)
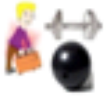	Heavy (Light)	C	128		heavy (50; 39%), difficult (5; 43%), heavier (4; 46%), struggle (3; 48%), strong (3; 51%), hard (3; 53%),
	Heavy (Light)	I	72	Struggle?	heavy (24; 33%), difficult (5; 40%), heavier (3; 44%), struggle (3; 49%)
	Heavy (Light)	V	56	none	heavy (26; 46%)

Note:
C = Combined Verbal first and Icon first groups.
I = Icon first group data only
V = Verbal first group data only
? = denotes count for word is close to criterion threshold (5)
none = no clearly cross-modal word reached criterion threshold (5)

Supplementary material (continued) for 'Verbal and Icon Ratings of Music Validation and Application of an Icon-Based Rating Scale'

DOI: 10.1177/2041669519852643

Schubert, E., Murari, M., Rodà, A., Canazza, S., Da Pos, O., & De Poli, G. (2019). Verbal and cross-modal ratings of music: validation and application of an icon-based rating scale. *i-Perception, 10(3),* 1–21. doi:10.1177/2041669519852643

Table 2. Ratings by item (verbal and icon) and music stimulus, highlighting items with large effect size.

Table 2. Ratings by item (verbal and icon) and music stimulus, highlighting items with large effect size.

Stimulus	Dominant pole[ad]	Mean[b]	|Cohen's—d|[c]	Item poles[d]
Bach		4.37 (1.58)	0.23	blue—orange
		3.94 (1.42)	0.04	Blue—Red Colour
		3.79 (1.55)	0.14	Cyan—Orange Colour
	active	2.54 (1.35)	1.08	active—passive
		4.95 (1.58)	0.60	calm—excited
		4.81 (1.5)	0.54	Calm—Excited Face
	happy	2.72 (1.41)	0.91	happy—sad
	Happy	2.68 (1.16)	1.13	Happy—Sad Face
	like	2.65 (1.55)	0.87	like—don't like
		4.23 (1.47)	0.16	Low—High Arousal SAM[e]
	Positive	2.81 (1.25)	0.95	Positive—Negative Valence SAM
	pleasant	2.35 (1.26)	1.31	pleasant—unpleasant
		3.85 (0.78)	0.19	scared—angry
		3.76 (0.81)	0.30	Scared—Angry Face
		3.39 (1.45)	0.42	strong—weak
		3.83 (1.49)	0.11	Submissive—Dominant SAM
		4.16 (1.57)	0.10	tense—relaxed
		3.97 (1.71)	0.02	Bouba—Kiki
		3.77 (1.43)	0.16	kiki—bouba
		3.98 (1.66)	0.01	Maluma—Takete
		4.1 (1.43)	0.07	maluma—takete
	Fast	2.65 (1.23)	1.10	Fast—Slow
		4.98 (1.39)	0.71	hard—soft
	light	5.68 (1.33)	1.26	heavy—light
		2.85 (1.58)	0.73	Light—Heavy
		3.47 (1.65)	0.32	Smooth—Rough
		3.02 (1.43)	0.68	smooth—rough
		3.19 (1.45)	0.56	Soft—Hard
	sweet	5.47 (1.21)	1.22	bitter—sweet
		4.17 (1.4)	0.12	Cold—Hot
		4.88 (1.38)	0.64	cold—warm

Note:
[a] Dominant pole shown for items with large effect size.
[b] N=144.
[c] |Cohen's—d| effect is shown for the mean rating with respect to scale centre (value of 4) where .2 is small .5 is medium .8 is large effect size. Items with large effect size are displayed with green background.
[d] Capitalised item pole labels refer to icon poles, all lower-case item poles refer to verbal pole labels. A selection at the leftmost pole was scored 1. A selection at the rightmost pole was scored 7.
[e] SAM = 'Self-Assessment Manikin' (Bradley & Lang, 1994).
Table 2 continues on the next page.

Stimulus	Dominant pole[ad]	Mean[b]	|Cohen's—d|[c]	Item poles[d]
Bizet		4.97 (1.5)	0.65	blue—orange
	Red Colour	5.1 (1.32)	0.83	Blue—Red Colour
	Orange Colour	5.1 (1.35)	0.82	Cyan—Orange Colour
	active	2.13 (1.12)	1.67	active—passive
	excited	5.88 (1.1)	1.72	calm—excited
		4.67 (1.6)	0.42	Calm—Excited Face
	happy	2.89 (1.24)	0.89	happy—sad
		3.04 (1.22)	0.78	Happy—Sad Face
		3.03 (1.41)	0.69	like—don't like
	High Arousal	5.33 (1.35)	0.99	Low—High Arousal SAM
		3.13 (1.34)	0.65	Positive—Negative Valence SAM
	pleasant	2.93 (1.29)	0.83	pleasant—unpleasant
		4.44 (0.94)	0.47	scared—angry
		4.51 (0.88)	0.58	Scared—Angry Face
	strong	2.07 (1.06)	1.83	strong—weak
		5.19 (1.49)	0.80	Submissive—Dominant SAM
	tense	2.7 (1.1)	1.19	tense—relaxed
	Kiki	5.42 (1.33)	1.07	Bouba—Kiki
		3.26 (1.28)	0.58	kiki—bouba
	Takete	5.35 (1.29)	1.04	Maluma—Takete
		4.47 (1.42)	0.33	maluma—takete
	Fast	2.35 (1.2)	1.38	Fast—Slow
	hard	2.85 (1.15)	1.00	hard—soft
		3.28 (1.42)	0.51	heavy—light
		4.67 (1.53)	0.44	Light—Heavy
		4.03 (1.68)	0.02	Smooth—Rough
		4.56 (1.37)	0.41	smooth—rough
		4.26 (1.41)	0.18	Soft—Hard
		4.23 (1.36)	0.17	bitter—sweet
	Hot	5.26 (1.34)	0.94	Cold—Hot
		4.94 (1.38)	0.68	cold—warm

Note:
[a] Dominant pole shown for items with large effect size.
[b] N=144.
[c] |Cohen's—d| effect is shown for the mean rating with respect to scale centre (value of 4) where .2 is small .5 is medium .8 is large effect size. Items with large effect size are displayed with green background.
[d] Capitalised item pole labels refer to icon poles, all lower-case item poles refer to verbal pole labels. A selection at the leftmost pole was scored 1. A selection at the rightmost pole was scored 7.
[e] SAM = 'Self-Assessment Manikin' (Bradley & Lang, 1994).
Table 2 continues on the next page.

Stimulus	Dominant pole[ad]	Mean[b]	|Cohen's—d|[c]	Item poles[d]
Brahms		3.63 (1.88)	0.20	blue—orange
		3.46 (1.61)	0.33	Blue—Red Colour
		3.19 (1.6)	0.51	Cyan—Orange Colour
	passive	5.41 (1.37)	1.03	active—passive
	calm	2.13 (1.23)	1.52	calm—excited
		3.92 (1.81)	0.04	Calm—Excited Face
		3.77 (1.73)	0.13	happy—sad
		3.65 (1.76)	0.20	Happy—Sad Face
		3.07 (1.6)	0.58	like—don't like
	Low	2.23 (1.11)	1.60	Low—High Arousal SAM
		3.6 (1.63)	0.25	Positive—Negative Valence SAM
	pleasant	2.26 (1.22)	1.43	pleasant—unpleasant
		3.77 (0.87)	0.27	scared—angry
		3.33 (0.89)	0.75	Scared—Angry Face
		4.68 (1.42)	0.48	strong—weak
		3.44 (1.6)	0.35	Submissive—Dominant SAM
	relaxed	5.86 (1.23)	1.52	tense—relaxed
	Bouba	2.07 (0.86)	2.25	Bouba—Kiki
		4.93 (1.44)	0.65	kiki—bouba
	Maluma	2.12 (0.99)	1.89	Maluma—Takete
		3.06 (1.39)	0.68	maluma—takete
	Slow	5.97 (1.04)	1.89	Fast—Slow
	soft	5.95 (1.09)	1.79	hard—soft
	light	5.76 (1.19)	1.48	heavy—light
	Light	2.44 (1.25)	1.25	Light—Heavy
	Smooth	2.58 (1.49)	0.95	Smooth—Rough
	smooth	2.03 (1.04)	1.89	smooth—rough
	Soft	2.17 (1.45)	1.27	Soft—Hard
	sweet	5.67 (1.23)	1.35	bitter—sweet
		3.28 (1.41)	0.51	Cold—Hot
		4.95 (1.61)	0.59	cold—warm

Note:
[a] Dominant pole shown for items with large effect size.
[b] N=144.
[c] |Cohen's—d| effect is shown for the mean rating with respect to scale centre (value of 4) where .2 is small .5 is medium .8 is large effect size. Items with large effect size are displayed with green background.
[d] Capitalised item pole labels refer to icon poles, all lower-case item poles refer to verbal pole labels. A selection at the leftmost pole was scored 1. A selection at the rightmost pole was scored 7.
[e] SAM = 'Self-Assessment Manikin' (Bradley & Lang, 1994).
Table 2 continues on the next page.

Stimulus	Dominant pole[ad]	Mean[b]	|Cohen's—d|[c]	Item poles[d]
Chopin		4.33 (1.84)	0.18	blue—orange
		5.1 (1.72)	0.64	Blue—Red Colour
		4.69 (1.76)	0.39	Cyan—Orange Colour
	active	2.44 (1.33)	1.18	active—passive
	excited	5.37 (1.28)	1.07	calm—excited
		3.2 (1.76)	0.45	Calm—Excited Face
		4.66 (1.02)	0.65	happy—sad
	Sad Face	5.04 (1.13)	0.92	Happy—Sad Face
		3.84 (1.73)	0.09	like—don't like
	High Arousal	5.69 (1.22)	1.39	Low—High Arousal SAM
		4.85 (1.54)	0.55	Positive—Negative Valence SAM
		4.38 (1.42)	0.27	pleasant—unpleasant
		4.83 (1.65)	0.50	scared—angry
		4.42 (1.85)	0.23	Scared—Angry Face
	strong	2.24 (1.17)	1.50	strong—weak
		5.1 (1.55)	0.71	Submissive—Dominant SAM
	tense	2.09 (1.14)	1.68	tense—relaxed
	Kiki	5.7 (1.29)	1.32	Bouba—Kiki
		3.24 (1.44)	0.53	kiki—bouba
	Takete	5.65 (1.3)	1.27	Maluma—Takete
		4.77 (1.36)	0.57	maluma—takete
		3.05 (1.91)	0.50	Fast—Slow
	hard	2.35 (1.03)	1.61	hard—soft
	heavy	2.48 (1.23)	1.24	heavy—light
	Heavy	5.6 (1.07)	1.49	Light—Heavy
	rough	5.44 (1.27)	1.13	smooth—rough
		4.66 (1.81)	0.37	Smooth—Rough
		4.86 (1.81)	0.48	Soft—Hard
	bitter	2.73 (1.01)	1.25	bitter—sweet
		5.1 (1.65)	0.67	Cold—Hot
		3.27 (1.58)	0.46	cold—warm

Note:
[a] Dominant pole shown for items with large effect size.
[b] N=144.
[c] |Cohen's—d| effect is shown for the mean rating with respect to scale centre (value of 4) where .2 is small .5 is medium .8 is large effect size. Items with large effect size are displayed with green background.
[d] Capitalised item pole labels refer to icon poles, all lower-case item poles refer to verbal pole labels. A selection at the leftmost pole was scored 1. A selection at the rightmost pole was scored 7.
[e] SAM = 'Self-Assessment Manikin' (Bradley & Lang, 1994).
Table 2 continues on the next page.

Stimulus	Dominant pole[ad]	Mean[b]	|Cohen's—d|[c]	Item poles[d]
Mozart	blue	2.33 (1.37)	1.22	blue—orange
	Blue	2.33 (1.32)	1.27	Blue—Red Colour
	Cyan	2.71 (1.52)	0.85	Cyan—Orange Colour
	passive	5.73 (1.12)	1.55	active—passive
	calm	1.99 (1.01)	2.00	calm—excited
	Calm	2.21 (1.39)	1.29	Calm—Excited Face
	sad	5.89 (1.4)	1.35	happy—sad
	Sad Face	5.8 (1.42)	1.27	Happy—Sad Face
	like	2.68 (1.51)	0.87	like—don't like
	Low	2.27 (1.38)	1.25	Low—High Arousal SAM
	Negative	5.63 (1.34)	1.21	Positive—Negative Valence SAM
		2.99 (1.48)	0.68	pleasant—unpleasant
		3.24 (1.06)	0.72	scared—angry
	Scared	2.44 (1.17)	1.33	Scared—Angry Face
		4.92 (1.56)	0.59	strong—weak
		3.05 (1.78)	0.54	Submissive—Dominant SAM
		5 (1.64)	0.61	tense—relaxed
	Bouba	2.33 (1.26)	1.32	Bouba—Kiki
		4.88 (1.37)	0.64	kiki—bouba
		3.12 (1.48)	0.59	maluma—takete
	Maluma	2.21 (1.2)	1.49	Maluma—Takete
	Slow	6.38 (0.76)	3.14	Fast—Slow
	soft	6.15 (0.96)	2.24	hard—soft
		5.15 (1.66)	0.69	heavy—light
		3.5 (1.92)	0.26	Light—Heavy
	smooth	1.98 (1.01)	1.99	smooth—rough
		2.99 (1.79)	0.57	Smooth—Rough
		3.76 (2.18)	0.11	Soft—Hard
		4.25 (1.71)	0.15	bitter—sweet
	Cold	2.45 (1.25)	1.24	Cold—Hot
		3.22 (1.76)	0.44	cold—warm

Note:
[a] Dominant pole shown for items with large effect size.
[b] N=144.
[c] |Cohen's—d| effect is shown for the mean rating with respect to scale centre (value of 4) where .2 is small .5 is medium .8 is large effect size. Items with large effect size are displayed with green background.
[d] Capitalised item pole labels refer to icon poles, all lower-case item poles refer to verbal pole labels. A selection at the leftmost pole was scored 1. A selection at the rightmost pole was scored 7.
[e] SAM = 'Self-Assessment Manikin' (Bradley & Lang, 1994).
Table 2 continues on the next page.

Stimulus	Dominant pole[ad]	Mean[b]	|Cohen's—d|[c]	Item poles[d]
Vivaldi		4.87 (1.48)	0.59	blue—orange
		4.03 (1.46)	0.02	Blue—Red Colour
		3.98 (1.52)	0.01	Cyan—Orange Colour
		3.01 (1.47)	0.67	active—passive
		4.76 (1.56)	0.49	calm—excited
	Excited Face	5.47 (1.28)	1.14	Calm—Excited Face
	happy	2.26 (1.01)	1.72	happy—sad
	Happy	2.24 (1.04)	1.69	Happy—Sad Face
		3.03 (1.4)	0.69	like—don't like
		3.99 (1.44)	0.01	Low—High Arousal SAM
	Positive	2.37 (1.18)	1.39	Positive—Negative Valence SAM
	pleasant	2.47 (1.26)	1.22	pleasant—unpleasant
		3.8 (0.6)	0.33	scared—angry
		3.92 (0.6)	0.13	Scared—Angry Face
		3.9 (1.24)	0.08	strong—weak
		3.91 (1.48)	0.06	Submissive—Dominant SAM
		4.77 (1.44)	0.54	tense—relaxed
		3.78 (1.6)	0.14	Bouba—Kiki
		3.67 (1.49)	0.22	kiki—bouba
		3.65 (1.55)	0.23	Maluma—Takete
		4.21 (1.41)	0.15	maluma—takete
		3.31 (1.29)	0.54	Fast—Slow
		4.98 (1.24)	0.79	hard—soft
	light	5.72 (1.08)	1.59	heavy—light
	Light	2.72 (1.41)	0.91	Light—Heavy
		3.47 (1.52)	0.35	Smooth—Rough
		3.22 (1.33)	0.58	smooth—rough
		2.96 (1.44)	0.72	Soft—Hard
	sweet	5.47 (1.07)	1.37	bitter—sweet
		4.19 (1.31)	0.15	Cold—Hot
	warm	5.35 (1.19)	1.13	cold—warm

Note:
[a] Dominant pole shown for items with large effect size.
[b] N=144.
[c] |Cohen's—d| effect is shown for the mean rating with respect to scale centre (value of 4) where .2 is small .5 is medium .8 is large effect size. Items with large effect size are displayed with green background.
[d] Capitalised item pole labels refer to icon poles, all lower-case item poles refer to verbal pole labels. A selection at the leftmost pole was scored 1. A selection at the rightmost pole was scored 7.
[e] SAM = 'Self-Assessment Manikin' (Bradley & Lang, 1994).
Table 2 continues on the next page.

Stimulus	Dominant pole[ad]	Mean[b]	|Cohen's—d|[c]	Item poles[d]
disliked piece (self-selected)		4.53 (1.81)	0.29	blue—orange
		4.92 (1.82)	0.51	Blue—Red Colour
		4.92 (1.65)	0.56	Cyan—Orange Colour
		3.07 (1.82)	0.51	active—passive
		4.91 (1.52)	0.60	calm—excited
		2.73 (1.78)	0.72	Calm—Excited Face
		3.97 (1.64)	0.02	happy—sad
		4.76 (1.77)	0.43	Happy—Sad Face
	don't like	6.67 (1)	2.68	like—don't like
		5.13 (1.87)	0.60	Low—High Arousal SAM
		5.06 (1.82)	0.58	Positive—Negative Valence SAM
	unpleasant	5.94 (1.45)	1.34	pleasant—unpleasant
		4.6 (1.44)	0.42	scared—angry
		5.16 (1.64)	0.71	Scared—Angry Face
		3.69 (2)	0.16	strong—weak
		4.5 (2.02)	0.25	Submissive—Dominant SAM
		3.15 (1.76)	0.48	tense—relaxed
		5.16 (1.86)	0.62	Bouba—Kiki
		3.51 (1.59)	0.31	kiki—bouba
		5.1 (1.86)	0.59	Maluma—Takete
		4.46 (1.54)	0.30	maluma—takete
		3.12 (1.75)	0.50	Fast—Slow
		3.09 (1.68)	0.54	hard—soft
		3.56 (1.88)	0.23	heavy—light
		4.9 (1.88)	0.48	Light—Heavy
		4.99 (1.94)	0.51	Smooth—Rough
		4.91 (1.77)	0.52	smooth—rough
		5.29 (1.7)	0.76	Soft—Hard
		3.11 (1.74)	0.51	bitter—sweet
		4.64 (1.88)	0.34	Cold—Hot
		3.65 (1.8)	0.19	cold—warm

Note:
[a] Dominant pole shown for items with large effect size.
[b] N=144.
[c] |Cohen's—d| effect is shown for the mean rating with respect to scale centre (value of 4) where .2 is small .5 is medium .8 is large effect size. Items with large effect size are displayed with green background.
[d] Capitalised item pole labels refer to icon poles, all lower-case item poles refer to verbal pole labels. A selection at the leftmost pole was scored 1. A selection at the rightmost pole was scored 7.
[e] SAM = 'Self-Assessment Manikin' (Bradley & Lang, 1994).
Table 2 continues on the next page.

Stimulus	Dominant pole[ad]	Mean[b]	|Cohen's—d|[c]	Item poles[d]
liked piece (self-selected)		4.17 (1.97)	0.09	blue—orange
		3.65 (1.74)	0.20	Blue—Red Colour
		4 (1.82)	0.00	Cyan—Orange Colour
		3.19 (1.88)	0.43	active—passive
		3.76 (2.25)	0.11	calm—excited
		5.01 (1.97)	0.51	Calm—Excited Face
		2.91 (1.81)	0.60	happy—sad
		2.64 (1.83)	0.74	Happy—Sad Face
	like	1.08 (0.3)	9.67	like—don't like
		3.51 (1.78)	0.28	Low—High Arousal SAM
	Positive	2.63 (1.7)	0.81	Positive—Negative Valence SAM
	pleasant	1.6 (1.01)	2.37	pleasant—unpleasant
		4.01 (0.6)	0.02	scared—angry
		3.65 (0.94)	0.37	Scared—Angry Face
	strong	2.43 (1.31)	1.20	strong—weak
		4.56 (1.72)	0.33	Submissive—Dominant SAM
		5 (1.86)	0.54	tense—relaxed
		3.38 (1.99)	0.31	Bouba—Kiki
		4.17 (1.61)	0.11	kiki—bouba
		3.24 (1.89)	0.40	Maluma—Takete
		3.69 (1.63)	0.19	maluma—takete
		4.28 (1.83)	0.15	Fast—Slow
		4.69 (1.78)	0.39	hard—soft
		4.52 (1.81)	0.29	heavy—light
		3.25 (1.78)	0.42	Light—Heavy
		3.19 (1.8)	0.45	Smooth—Rough
	smooth	2.6 (1.7)	0.82	smooth—rough
	Soft	2.6 (1.64)	0.85	Soft—Hard
	sweet	5.34 (1.55)	0.86	bitter—sweet
		4.42 (1.69)	0.25	Cold—Hot
	warm	5.49 (1.54)	0.97	cold—warm

Note:
[a] Dominant pole shown for items with large effect size.
[b] N=144.
[c] |Cohen's—d| effect is shown for the mean rating with respect to scale centre (value of 4) where .2 is small .5 is medium .8 is large effect size. Items with large effect size are displayed with green background.
[d] Capitalised item pole labels refer to icon poles, all lower-case item poles refer to verbal pole labels. A selection at the leftmost pole was scored 1. A selection at the rightmost pole was scored 7.
[e] SAM = 'Self-Assessment Manikin' (Bradley & Lang, 1994).
